# Cigarette Use, Lung Cancer Screening Eligibility and Completion Among Persons With Poor Mental Health

**DOI:** 10.1002/cam4.70983

**Published:** 2025-05-30

**Authors:** Monica Hernandez, Anastasia Rogova, Lorraine R. Reitzel, Lisa M. Lowenstein, Robert J. Volk

**Affiliations:** ^1^ Health Services Research (HSR) Department The University of Texas MD Anderson Cancer Center Houston Texas USA; ^2^ Behavioral Science Department The University of Texas MD Anderson Cancer Center Houston Texas USA

**Keywords:** cigarette use, depression, early detection of cancer, lung cancer, mental distress

## Abstract

**Background:**

Depression and mental distress are associated with greater cigarette use; however, it remains unclear how poor mental health relates to eligibility and completion of lung cancer screening (LCS).

**Methods:**

Study of a 2022 Behavioral Risk Factor Surveillance System (BRFSS) sample of adults aged 50+. Key mental health exposures for this paper were (i) any history of a depressive disorder, and (ii) frequent mental distress (FMD) in the last month. Descriptive analyses were conducted on all variables and ran separately on each mental health exposure to explore associations between mental health conditions, cigarette use, LCS eligibility, and completion.

**Results:**

Compared to adults without a depressive history, adults with a depressive history were more likely to currently smoke (19.9% vs. 10.8%); have a slightly higher average pack‐year history (26.8 vs. 24.0 years); and were more likely to be eligible for LCS (18.9% vs. 10.8%). Among adults eligible for LCS, there was no difference in completion of screening in the last year between adults with versus without a depressive history (19.4% vs. 18.7%). A similar pattern of findings was observed for people with and without FMD.

**Conclusions:**

Cigarette use is more common among persons with a history of depression or FMD, yet they are screened for lung cancer at similar rates compared to their counterparts without a history of depression or FMD. LCS rates are also low among persons with poor mental health, mirroring screening among general U.S. adults. Mechanisms to increase LCS rates among adults with mental health conditions are discussed.

## 1 Introduction

Lung cancer remains the leading cause of cancer‐related mortality in the United States, with 127,070 estimated deaths in 2023 [1]. Smoking cessation is the main way to prevent lung cancer among adults who smoke conventional cigarettes, whereas advances in early detection via low‐dose computed tomography (LDCT) allow for the diagnosis of lung cancer at an earlier and more treatable stage, contributing to decreased mortality [2]. Rates of LDCT lung cancer screening (LCS) are low, estimated at about 16% among LCS‐eligible adults aged 50+ years [3]. Multiple factors may account for the low completion of LCS, including patients' reported fear of the diagnosis and treatment [4], limited knowledge of the screening availability [5, 6, 7], concerns about the test [8], and practical barriers [9]. Largely uneven rates of screening completion are observed among eligible White and Black individuals (about 16.1% vs. 21.0%, respectively), as well as males and females (about 19.9% vs. 22.6%, respectively) in the United States [3, 10, 11]. The social determinants of health, such as inequities in healthcare access—including being uninsured or underinsured and belonging to disadvantaged groups of low socioeconomic status (SES)—can present additional cost and access‐related barriers to LCS among marginalized adults [10, 12, 13, 14, 15, 16]. A history of mental illness is another factor associated with a limited access to healthcare, including cancer screening [17, 18, 19]. However, this association has been insufficiently addressed in LCS research, with only limited data suggesting lower rates of LCS among this group, resulting in the need for additional research [20].

An existing gap remains in understanding how mental health conditions affect LCS completion as individuals with mental health conditions have significantly higher cigarette smoking rates when compared to the general population [21, 22]. Smoking rates among this group are between 30%–70% [23, 24]; and up to 48% among those with moderate or severe depressive symptoms [22, 25], versus about 11.6% in the general population. High rates of cigarette smoking place individuals with mental health conditions at a higher risk of developing lung cancer. Prior research also indicates that individuals with mental health conditions are less likely to receive screening for any type of cancer, with most research focusing on breast, colon, and cervical cancers [20, 26, 27]. The data on LCS rates among eligible individuals with mental health conditions are limited; however, they might mirror the lower rates of screening for other cancers [20, 28]. This disparity may also be associated with other factors, including stigmatizing attitudes toward mental health conditions, and further exacerbated by stigma surrounding smoking and smoking‐related illness [29].

The current study explores associations between mental health conditions and cigarette use, LCS eligibility and completion. Using data from the 2022 Behavioral Risk Factor Surveillance System (BRFSS) cross‐sectional dataset, we specifically address the following four research questions of interest: “Are adults reporting history of depression and/or mental distress more or less likely than their counterparts to (i) currently smoke cigarettes; (ii) have a higher pack‐year history; (iii) be more likely to be eligible for LCS; and (iv) be more or less likely to complete LCS in the last year?” Prior studies have used BRFSS data to identify factors associated with LCS completion, including insurance, age, marital status, prior cancer diagnosis, COPD, having a primary care provider [30, 31, 32]. However, there are no studies, to our knowledge, that have addressed mental health status as a factor associated with LCS eligibility and completion. Inasmuch as inequities may exist, or low rates of LCS completion are found overall, results may suggest the need for more targeted efforts to increase LCS among adults with a mental illness.

## 2 Methods

### 2.1 Data Sources and Variables

Data for this analysis come from the 2022 Behavioral Risk Factor Surveillance System (BRFSS) cross‐sectional dataset [33]. BRFSS is a state‐based surveillance system administered by the Population Health Surveillance Branch under the Center for Disease Control (CDC). Since 1984, BRFSS has conducted monthly health‐related telephone interviews among noninstitutionalized U.S. adults aged 18+ years across all 50 U.S. states. Using an independent probability sampling method, the BRFSS core questionnaire collects respondent information on self‐reported health‐related risk behaviors, chronic health conditions, healthcare access, and use of preventive services.

Key variables for this analysis included LCS eligibility and completion status as well as cigarette use variables. As per 2021 USPSTF‐LCS guidelines, LCS eligibility status was defined from BRFSS questions assessing (i) respondents aged 50–80 years old who (ii) currently smoke cigarettes or have quit within the last 15 years and (iii) have a 20+ pack‐year of smoking history [34]. LCS completion status was derived from questions assessing eligible respondents who received a CAT/CT scan of the chest for lung cancer in the last year. Respondents were prompted with the following statement, “The next question is about CT or CAT scans of your chest area. During this test, you lie flat on your back and are moved through an open, donut shaped x‐ray machine”. Respondents were then asked if and when the CT or CAT scans of the chest area were done to check or screen for lung cancer in the following series of questions: (i) “Have you ever had a CT or CAT scan of your chest area?” (Y/N); (ii) “Were any of the CT or CAT scans of your chest area done mainly to check or screen for lung cancer?” (Y/N); (iii) “When did you have your most recent CT or CAT scan of your chest area mainly to check or screen for lung cancer?” (< 1/1+ year). Binary responses (Y/N) were defined to distinguish LCS eligible respondents who also completed LCS in the last year (Y/N). Variables related to cigarette use were smoking status (currently smoke/formerly smoke/never smoke) and pack‐year history (# years).

Key mental health variables for this analysis were lifetime history of a depressive disorder and frequent mental distress (FMD) in the last month. History of a depressive disorder was self‐assessed by the question asking, “(Ever told) (you had) a depressive disorder (including depression, major depression, dysthymia, or minor depression)?”. Binary responses (Y/N) distinguished respondents with and without a history of depressive disorder. Frequent mental distress (FMD) was also self‐assessed by the question asking, “Now thinking about your mental health, which includes stress, depression, and problems with emotions, for how many days during the past 30 days was your mental health not good?” Binary responses (0–13/14–30 days) distinguished respondents with and without poor mental health. Respondents reporting 14–30 days of poor mental health were categorized as having FMD [35]. Standard demographic and socioeconomic indicators were also included in the analysis, including age, sex, race/ethnicity, health insurance type, annual income and education level. We also included the BRFSS calculated pack‐year variable which is defined as the self‐reported number of years smoking cigarettes multiplied by reported packs smoked per day.

Note: Results from survey‐weighted preliminary analyses revealed that our mental health variables were both lowly correlated (*R* = 0.37; *p* < 0.001) and statistically different (Pearson's *χ* ^2^, *p* < 0.001). Therefore, in this analysis, we ran these variables separately as each captured a distinct mental health construct and each had a different timeframe of relevance; history of depressive disorder captured any depressive disorder over the respondent's lifetime, whereas FMD captured the respondent's general distress over the last month. Related studies conducted on BRFSS samples by Rashid et al. (2022) and Mojtabai et al. (2015) have also examined FMD and depressive disorders as separate constructs [36, 37].

Despite not having much statistical significance, we acknowledge the clinical or real‐world significance of examining adults presenting with either versus neither mental health conditions. Therefore, in additional analyses [*not shown*], we examined associations across all study variables among respondents reporting having either versus neither FMD and/or a depressive disorder history. Trends from this analysis (i.e., *p* values and frequency differences) were largely similar to trends observed in main analyses in which FMD and depressive disorder history were separately examined.

### 2.2 Statistical Analyses

This analysis was restricted to adults aged 50–79 years. BRFSS collapses responses for adults aged 80+ years, which is the upper age limit under 2021 USPSTF‐LCS guidelines [34]. We therefore included respondents up to age 79. Weighted and unweighted descriptive analyses (Tables 1, 2, 3) were initially conducted for all variables and then separately on each mental health exposure (weighted only). All descriptive analyses were estimated using the Pearson's chi‐square test for categorical variables and ANOVA test for continuous variables. Weighting for this analysis was based on the BRFSS methodology of iterative proportional fitting (or “raking”), which ensures a more accurate reflection of the sociodemographic makeup of individual states [33].

**TABLE 1:** Descriptive sample characteristics, BRFSS 2022 sample aged 50–79 years (Unweighted & weighted).

Variable	Descriptive statistic (*n* [%]; mean [SD])	
	Unweighted (*N* = 115,292)	Weighted (*N* = 56,241,409)
Age		
50–64 years	56,607 (49.1%)	32,470,984 (57.7%)
65–79 years	58,685 (50.9%)	23,770,425 (42.3%)
Sex		
Male	52,566 (45.6%)	26,551,350 (47.2%)
Female	62,726 (54.4%)	29,690,059 (52.8%)
Race/ethnicity		
Hispanic	5675 (5.1%)	7,025,887 (12.9%)
NHB	10,873 (9.8%)	6,494,057 (11.9%)
NHM	2025 (1.8%)	1,414,693 (2.6%)
NHO^d^	4303 (3.9%)	3,851,787 (7.1%)
NHW	88,556 (79.5%)	35,564,167 (65.4%)
Health insurance^e^		
Private	44,619 (40.0%)	23,930,079 (44.2%)
Public	63,589 (57.1%)	28,021,285 (51.8%)
None	3227 (2.9%)	2,188,162 (4.0%)
Income		
< $25 K	14,569 (16.1%)	7,471,626 (17.2%)
$25 K– $49,999	22,640 (25.0%)	10,514,296 (24.3%)
$50 K–$74,999	15,933 (17.6%)	6,847,308 (15.8%)
$75 K–$99,999	12,674 (14.0%)	5789,903 (13.4%)
$100 K+	24,711 (27.3%)	12,711,686 (29.3%)
Education		
Did not graduate HS	5789 (5.1%)	6,198,703 (11.1%)
Graduated HS	26,625 (23.2%)	13,770,157 (24.7%)
Some college or technical school	31,909 (27.8%)	17,847,679 (32.0%)
Graduated from college	50,280 (43.9%)	18,026,537 (32.3%)
Depressive disorder history		
Yes	21,769 (19.0%)	10,166,583 (18.2%)
No	92,950 (81.0%)	45,799,134 (81.8%)
Frequent mental distress (FMD)		
Yes^b^	12,963 (11.2%)	6,705,869 (11.9%)
No	102,327 (88.8%)	49,534,466 (88.1%)
Eligible for LCS^c^		
Yes	13,268 (12.5%)	6,286,297 (12.3%)
No	92,917 (87.5%)	44,735,444 (87.7%)
Screened for lung cancer within last year, among eligible^c^		
Yes	2331 (19.3%)	1,078,934 (18.9%)
No	9736 (80.7%)	4,640,110 (81.1%)
Smoking status		
Currently smokes, smokes daily	9780 (9.2%)	4,722,211 (9.3%)
Currently smokes, smokes some days	3267 (3.1%)	1,668,209 (3.3%)
Formerly smoked	33,915 (31.9%)	15,656,428 (30.7%)
Never smoked	59,223 (55.8%)	28,974,892 (56.8%)
Pack‐year history^a^		
Mean [SD]	25.1 [26.2]	24.7 [25.5]
Median [IQR]	19 [29]	18 [29]
Cigarettes per day, lifetime smokers		
Mean [SD]	16.7 [13.5]	16.3 [13.8]
Median [IQR]	15 [10]	15 [12]

Abbreviations: IQR, interquartile range; LCS, lung cancer screening; NHB, non‐Hispanic Black; NHM, non‐Hispanic Multiracial; NHO, non‐Hispanic Other; NHW, non‐Hispanic White; SD, standard deviation.

^a^ Pack‐year history calculated among current and former smokers.

^b^ Respondents reporting 14–30 days of poor mental health are defined as having frequent mental distress (FMD).

^c^ As per 2021 USPSTF‐LCS eligibility guidelines.

^d^ NHO: non‐Hispanic American Indian or Alaska Native, Asian, Native Hawaiian or Other Pacific Islander.

^e^ Private: Employer or private NGO; Public: Medicare, Medigap, Medicaid, CHIP, Military, Indian Health Service, other gov't.

**TABLE 2:** Bivariable analysis of depressive disorder history and study variables, BRFSS 2022 sample aged 50–79 years (weighted).

Variable	Depressive disorder history [*n* (%); mean (SD)] (*N* = 55,965,717)		
	No (*N* = 45,799,134)	Yes (*N* = 10,166,583)	*p‐value* ^a^ (Pearson's *χ* ^2^; ANOVA test)
Age			
50–64 years	25,993,297 (56.8%)	6,312,170 (62.1%)	< 0.001
65–79 years	19,805,838 (43.2%)	3,854,413 (37.9%)	
Sex			
Male	23,031,003 (50.3%)	3,385,327 (33.3%)	< 0.001
Female	22,768,131 (49.7%)	6,781,255 (66.7%)	
Race/ethnicity			
Hispanic	5,872,473 (13.3%)	1,132,679 (11.5%)	< 0.001
NHB	5,479,751 (12.4%)	984,343 (10.0%)	
NHM	978,228 (2.2%)	430,087 (4.4%)	
NHO^b^	3,457,954 (7.8%)	376,160 (3.8%)	
NHW	28,479,344 (64.3%)	6,952,403 (70.4%)	
Health insurance^c^			
Private	20,489,282 (46.5%)	3,366,730 (34.3%)	< 0.001
Public	21,798,562 (49.4%)	6,093,062 (62.0%)	
None	1,797,445 (4.1%)	369,782 (3.8%)	
Income			
<$25 K	5,088,423 (14.5%)	2,327,721 (28.7%)	< 0.001
$25 K to $49,999	8,366,474 (23.8%)	2,109,691 (26.0%)	
$50 K to $74,999	5,699,009 (16.2%)	1,129,662 (13.9%)	
$75 K to $99,999	4,868,802 (13.9%)	911,979 (11.2%)	
$100 K+	11,064,737 (31.5%)	1,629,671 (20.1%)	
Education			
Did not graduate HS	4,685,299 (10.3%)	1,481,817 (14.6%)	< 0.001
Graduated HS	11,451,397 (25.2%)	2,272,296 (22.4%)	
Some college or technical school	14,090,951 (31.0%)	3,676,341 (36.3%)	
Graduated from college	15,268,006 (33.6%)	2,692,564 (26.6%)	
Eligible for LCS			
Yes	4,477,290 (10.8%)	1,777,909 (18.9%)	< 0.001
No	36,949,828 (89.2%)	7,618,485 (81.0%)	
Screened for lung cancer within last year, among eligible			
Yes	763,278 (18.7%)	311,061 (19.4%)	0.65
No	3,326,850 (81.3%)	1,291,430 (80.6%)	
Smoking status			
Currently smokes	4,490,474 (10.8%)	1,867,890 (19.9%)	< 0.001
Formerly smoked	12,300,922 (29.7%)	3,289,623 (35.0%)	
Never smoked	24,635,723 (59.5%)	4,238,883 (45.1%)	
Pack‐year history			
Mean [SD]	24.0 [25.5]	26.8 [25.5]	< 0.001
Median [IQR]	17 [29]	20 [30]	
Cigarettes per day, lifetime smokers			
Mean [SD]	16.1 [14.0]	16.8 [13.1]	< 0.001
Median [IQR]	15 [12]	15 [10]	

Abbreviations: IQR, interquartile range; LCS, lung cancer screening; NHB, non‐Hispanic Black; NHM, non‐Hispanic Multiracial; NHO, non‐Hispanic Other; NHW, non‐Hispanic White; SD, standard deviation.

^a^ *p* values were generated using Pearson's *χ* ^2^ for categorical variables and ANOVA test for continuous variables (on means).

^b^ NHO: non‐Hispanic American Indian or Alaska Native, Asian, Native Hawaiian or Other Pacific Islander.

^c^ Private: Employer or private NGO; Public: Medicare, Medigap, Medicaid, CHIP, Military, Indian Health Service, other gov't.

**TABLE 3:** Bivariable analysis of frequent mental distress (FMD) over last month and study variables, BRFSS 2022 sample aged 50–79 years (weighted).

Variable	Frequent mental distress (FMD)^b^ [*n* (%); mean (SD)] (*N* = 56,240,335)		
	No (*N* = 49,534,466)	Yes (*N* = 6,705,869)	*p‐value* ^a^ (Pearson's *χ* ^2^; ANOVA test)
Age			
50–64 years	28,100,269 (56.7%)	4,369,640 (65.2%)	< 0.001
65–79 years	21,434,197 (43.3%)	2,336,228 (34.84%)	
Sex			
Male	23,961,564 (48.4%)	2,589,786 (38.62%)	< 0.001
Female	25,572,902 (51.6%)	4,116,082 (61.38%)	
Race/ethnicity			
Hispanic	6,131,747 (12.8%)	894,140 (13.8%)	< 0.01
NHB	5,652,166 (11.8%)	841,891 (13.0%)	
NHM	1,166,426 (2.4%)	248,266 (3.8%)	
NHO^c^	3,469,620 (7.2%)	382,167 (5.9%)	
NHW	31,472,220 (65.7%)	4,091,946 (63.4%)	
Health insurance^d^			
Private	21,808,710 (45.7%)	2,121,369 (33.0%)	< 0.001
Public	24,126,512 (50.6%)	3.894,772 (60.6%)	
None	1,773,428 (3.7%)	414,734 (6.4%)	
Income			
< $25 K	5,651,661 (14.8%)	1,819,966 (34.7%)	< 0.001
$25 K to $49,999	9,095,898 (23.9%)	1,418,398 (27.1%)	
$50 K to $74,999	6,171,148 (16.2%)	676,160 (12.9%)	
$75 K to $99,999	5,311,397 (13.9%)	478,427 (9.1%)	
$100 K+	11,863,474 (31.1%)	848,212 (16.2%)	
Education			
Did not graduate HS	4,948,498 (10.1%)	1,250,206 (18.8%)	< 0.001
Graduated HS	12,009,146 (24.4%)	1,761,011 (26.4%)	
Some college or technical school	15,590,331 (31.7%)	2,257,348 (33.9%)	
Graduated from college	16,633,123 (33.8%)	1,393,414 (20.9%)	
Eligible for LCS			
Yes	4,958,935 (11.0%)	1,327,362 (21.7%)	< 0.001
No	39,932,049 (89.0%)	4,803,395 (78.3%)	
Screened for lung cancer within last year, among eligible			
Yes	851,331 (18.8%)	227,603 (19.2%)	0.81
No	3,683,943 (81.2%)	956,167 (80.8%)	
Smoking status			
Currently smokes	4,902,901 (10.9%)	1,487,520 (24.3%)	< 0.001
Formerly smoked	13,552,590 (30.2%)	2,103,838 (34.3%)	
Never smoked	26,435,494 (58.9%)	2,539,398 (41.4%)	
Pack‐year history			
Mean [SD]	24.1 [25.7]	27.3 [24.4]	< 0.001
Median [IQR]	17 [28]	21 [30]	
Cigarettes per day, lifetime smokers			
Mean [SD]	16.3 [14.1]	16.4 [12.5]	< 0.001
Median [IQR]	15 [12]	15 [13]	

Abbreviations: IQR, interquartile range; LCS, lung cancer screening; NHB, non‐Hispanic Black; NHM, non‐Hispanic Multiracial; NHO, non‐Hispanic Other; NHW, non‐Hispanic White; SD, standard deviation.

^a^ *p* values were generated using Pearson's *χ* ^2^ for categorical variables and ANOVA test for continuous variables (on means).

^b^ Respondents reporting 14–30 days of poor mental health are defined as having frequent mental distress (FMD).

^c^ NHO: non‐Hispanic American Indian or Alaska Native, Asian, Native Hawaiian or Other Pacific Islander.

^d^ Private: Employer or private NGO; Public: Medicare, Medigap, Medicaid, CHIP, Military, Indian Health Service, other gov't.

Given the related literature on disparities in healthcare access among at‐risk populations, we conducted additional sensitivity analyses of weighted logistic regression models to evaluate the extent to which the association between mental health status and LCS completion differed by sex and race/ethnicity (Tables S1a–S4a). We also conducted sensitivity analyses replacing pack‐year history with cigarettes per day in stratified regression models (Tables S1b–S4b), as per preliminary analyses indicating a high correlation between these variables (survey‐weighted *R* = 0.79; *p* < 0.001). Regression models included odds ratios (OR) and 95% confidence intervals. Alpha values of *a* = 0.05, 0.01, and 0.001 were used as statistically significant cutoffs for multiple tests. *p* values were two‐sided, indicated as (*) *p* ≤ 0.05; (**) *p* ≤ 0.01; (***) *p* ≤ 0.001. All missing data was excluded from this analysis, which included responses indicated as “excluded” and “refused”. Multiple imputation was not performed as sample sizes remained large enough to attain a reasonable analytical power level. This study was exempt from the Institutional Review Board at the University of Texas MD Anderson Cancer Center as per the Common Rule for publicly available data [38]. STATA/SE version 18.0 was used for all analyses [39].

## 3 Results

Table 1 presents weighted and unweighted descriptive characteristics for our sample. Weighted data indicate that our overall sample (*n* = 56,241,409) primarily consisted of adults aged 50–64 years (57.7%), females (52.8%), and persons identifying as non‐Hispanic White (NHW; 65.4%). Roughly 18% of adults in our sample reported having any history of a depressive disorder, whereas 11.9% reported FMD in the last month. The median pack‐year history among adults who currently smoked was 24.7 pack‐years, whereas the median cigarettes smoked per day was 16.3 cigarettes. Among 12.3% of LCS‐eligible adults in our sample, only 18.9% completed LCS in the last year.

Tables 2 and 3 present weighted descriptive characteristics for our sample by depressive disorder history and FMD in the last month, respectively. Results from Table 2 indicate that, compared to adults without a depressive history, adults with a depressive history were more likely to currently smoke (19.9% vs. 10.8%); have a slightly higher average pack‐year history (26.8 vs. 24.0 years); and be more likely to be eligible for LCS (18.9% vs. 10.8%). Among adults eligible for LCS, there was no difference in completion of screening in the last year between adults with versus without a depressive history (19.4% vs. 18.7%). Similarly, results from Table 3 indicate that, compared to adults without FMD, adults with FMD were more likely to currently smoke (10.9% vs. 24.3%); have a slightly higher average pack‐year history (24.1 vs. 27.3 years); and be eligible for LCS (11.0% vs. 21.7%). Among LCS eligible adults, the likelihood of screening completion in the last year was similar among adults with and without FMD (19.2% vs. 18.8%).

Sensitivity Tables S1a–S4a indicate results from weighted logistic regression models stratified by both sex and race/ethnicity for each mental health exposure. Overall associations between depressive disorder history/FMD and screening completion were positive yet nonsignificant (respectively, OR = 1.15 [0.90–1.47]; OR = 1.14 [0.87–1.48]). Among other covariates, overall odds of screening completion across both depressive disorder history/FMD models were significantly higher among adults aged 65–79 vs. 50–64 years (1.99 < OR < 2.04) and those with an increased pack‐year history (OR = 1.01). Conversely, overall screening completion odds were significantly lower among self‐identifying non‐Hispanic Multiracial (NHM) versus NHW adults (0.47 < OR < 0.49) and those with no versus private health insurance (OR = 0.24). Associations between mental health conditions and screening completion varied by sex and race/ethnicity; however, sex/race‐specific interaction terms in respective models were generally nonsignificant. Moreover, the magnitude and direction of results from sensitivity models replacing pack‐year history with cigarette use (Tables S1b–S4b) did not vary.

## 4 Discussion

In this analysis of a nationally representative sample of U.S. adults in 2022, we found that adults with a depressive disorder history/FMD were more likely to currently smoke, have a slightly higher average pack‐year history, and were more likely to be eligible for LCS than their counterparts without a depressive disorder history/FMD. We also found that the likelihood of screening completion in the last year among LCS eligible adults was similar among adults with and without a depressive disorder history/FMD but was lower than desirable in both groups, suggesting the need for interventions to increase LCS uptake.

Findings from our sample indicated higher average pack‐year history as well as current smoking rates that were nearly two times higher among adults with versus without a depressive disorder history (19.9% vs. 10.8%); and FMD (24.3% vs. 10.9%). Such results align with findings from other nationally representative studies including a National Surveys on Drug Use and Health (NSDUHs) study of older adults aged 50+ years indicating higher rates of mental illness among adults who currently smoke versus do not smoke (25.2% vs. 14.9%), including a slightly higher average number of cigarettes smoked in the last month among adults with versus without any mental illness (respectively, 396 vs. 364) [21, 23, 40]. It is also the case that persons with mental health conditions are often not screened for tobacco use or receive cessation/treatment services in the settings where they receive care [41]. There is a dual missed opportunity related to both failing to screen for tobacco use, the primary risk factor for lung cancer, and support screening for lung cancer [42].

A similar likelihood of screening completion in the last year among adults in our sample with and without a depressive disorder history (19.4% vs. 18.7%) and FMD (19.2% vs. 18.8%) contrasts with extant literature indicating lower cancer screening completion rates among adults with mental health conditions [20]. For instance, using administrative claims data, a study by Murphy et al. (2021) reported that persons with serious mental illness (SMI), specifically schizophrenia or bipolar disorder, were respectively less likely than persons without SMI to receive screening for four different cancer types including cervical (42% vs. 49%), breast (43% vs. 51%), colorectal (18% vs. 19%), and prostate (39% vs. 44%) cancer screenings [43]. Clinicians interviewed for that study attributed five central barriers, namely, access to care, available support, prioritization of other issues, communication, and patient concerns as reasons for the screening disparity. However, differences in results from that work and the current study may be explained by variations in mental health variable definitions and prevalence (e.g., ≈6% SMI from national estimates [44] vs. ≈18% lifetime depressive disorder in this study).

We found that LCS completion rates among adults with mental health conditions in this study (roughly 19%) are generally similar to those from nationally representative samples of the general population. BRFSS data from 2022 indicated LCS rates of 18% among eligible adults, while the 2021 BRFSS indicated LCS rates of 21% among eligible adults [45, 46]. Differences in sample selection criteria as well as applications of different LCS eligibility criteria in these studies (i.e., 2013 vs. 2021 USPSTF criteria) may help explain slight variations in reported LCS rates across these studies and our own. Although LCS completion rates among eligible adults are not ideal overall, adults with mental health conditions display LCS completion rates that are generally lower than other cancer screening types (i.e., approximately 40%), as per Murphy et al. [43].

### 4.1 Limitations

This study had several limitations. First, we were unable to draw temporal inferences due to the cross‐sectional nature of our dataset. We were therefore unable to determine directionality as per studies reporting reverse associations between LCS and psychological distress [47]. Future studies might draw from longitudinal datasets to evaluate causal associations between factors related to mental health status and LCS eligibility and completion, as well as individual changes in LCS eligibility and completion among adults who no longer report FMD within the last month. Second, due to dataset limitations, we did not include other LCS eligibility criteria, such as additional co‐morbidities, as well as potential confounders of screening completion such as diagnosable mental health disorders, fear of diagnosis and treatment, limited knowledge of the screening availability, and concerns about the test [48]. Third, our analysis was subject to reporting bias as LCS status and other health‐related measures were self‐reported.

### 4.2 Implications and Conclusions

Mental health conditions present a complex set of challenges for individuals and their healthcare providers, including increased incidence of physical illness and premature mortality [49, 50]. By evaluating connections between mental health conditions and cigarette use, LCS eligibility/completion trends, we aim to encourage the development of targeted interventions to improve rates of LCS completion and contribute to better physical health outcomes for people living with mental health conditions. Multilevel interventions that address the intersectionality of health inequities and what contributes to them (e.g., social determinants of health, lack of universal screening for smoking history in behavioral health settings) need to be developed and promoted to boost LCS completion among eligible patients (e.g., persons of low SES, minoritized adults) with mental health conditions who face increased cost and access‐related barriers to healthcare access [28, 51]. One approach is to integrate LCS facilitation into behavioral healthcare settings where individuals with mental health conditions receive care [42]. Potential benefits to this model include more regular contact and a heightened level of trust between mental health providers and their patients, as well as a better understanding of patients' needs and potential barriers to screening. Another approach might include the expansion of the Comprehensive Primary Care (CPC) initiative and related primary care medical home (PMCH) model, for which the Centers for Medicare & Medicaid Services (CMS) would reimburse participating primary care sites engaging in its core functions including risk stratified care management, planned care for chronic conditions and preventive care, and coordination of care across the medical neighborhood [52]. Expansion of the CPC initiative could help address relevant healthcare barriers among at‐risk adults with mental health conditions (e.g., cognitive deficits, patient‐provider communication challenges, and fragmented primary care and mental health care), and further promote collaborations between patients, providers, and the community to co‐design relevant LCS messaging and outreach efforts [53, 54].

## Author Contributions

**Monica Hernandez:** conceptualization (equal), data curation (lead), formal analysis (lead), investigation (lead), methodology (equal), writing – original draft (lead), writing – review and editing (lead). **Anastasia Rogova:** conceptualization (equal), methodology (equal), writing – original draft (supporting), writing – review and editing (supporting). **Lorraine R. Reitzel:** conceptualization (equal), methodology (equal), writing – review and editing (supporting). **Lisa M. Lowenstein:** conceptualization (equal), data curation (supporting), methodology (equal), writing – review and editing (supporting). **Robert J. Volk:** conceptualization (equal), funding acquisition (supporting), methodology (equal), supervision (lead), writing – original draft (supporting), writing – review and editing (supporting).

## Conflicts of Interest

Dr. Monica Hernandez is supported by the annual distributions of the Permanent Health Fund endowment received by the University of Texas MD Anderson Cancer Center from the state legislature. No other disclosures are reported. The funders of this study did not play a role in the design of the study; the collection, analysis, and interpretation of the data; the writing of the manuscript; and the decision to submit the manuscript for publication.

## Supporting information


Table S1

## Data Availability

The data that support the findings of this study are openly available in the 2022 BRFSS Survey Data and Documentation at https://www.cdc.gov/brfss/annual_data/annual_2022.html. The authors confirm that the data supporting the findings of this study are available within the article and/or its Supporting Information. Additional requests for variable datasets and statistical analyses used in this study may be sent to Dr. Robert J. Volk at bvolk@mdanderson.org.
