# Supplementary material for: Cigarette Use, Lung Cancer Screening Eligibility and Completion Among Persons With Poor Mental Health
Source: Cancer Med. 2025 May 30;14(11):e70983. doi: 10.1002/cam4.70983 (PMC12123384; doi:10.1002/cam4.70983)
Supplement: Supplementary file 1 — Table S1 [file CAM4-14-e70983-s001.docx]

**e-Tables**

e-Table 1a: Logistic regressions predicting associations between depressive disorder history & other covariates/pack-year history on LCS overall and by sex, BRFSS 2022 sample aged 50-79 years (Weighted)

| Variable | Odds of LCS [Odds ratio (95%CI)] ^a^ | | |
| --- | --- | --- | --- |
|  | Overall  (N=4,641,820) | Male  (N=2,598,051) | Female  (N=2,043,769) |
| Depressive disorder history |  |  |  |
| Yes | 1.15 (0.90 to 1.47) | 0.97 (0.66 to 1.41) | 1.33 (0.98 to 1.80) |
| No (Ref) |  |  |  |
|  |  |  |  |
| Age |  |  |  |
| 50-64 years (Ref) |  |  |  |
| 65-79 years | 2.04 (1.63 to 2.55)*** | 2.27 (1.65 to 3.13)*** | 1.78 (1.34 to 2.37)*** |
|  |  |  |  |
| Sex |  |  |  |
| Male (Ref) |  |  |  |
| Female | 0.98 (0.80 to 1.21) | -- | -- |
|  |  |  |  |
| Race/ethnicity |  |  |  |
| Hispanic | 0.81 (0.42 to 1.55) | 0.87 (0.40 to 1.91) | 0.67 (0.23 to 1.95) |
| NHB | 1.09 (0.74 to 1.62) | 0.99 (0.61 to 1.59) | 1.30 (0.67 to 2.54) |
| NHM | 0.47 (0.27 to 0.81)** | 0.52 (0.26 to 1.07) | 0.37 (0.16 to 0.86)* |
| NHO ^b^ | 1.85 (0.88 to 3.90) | 1.29 (0.46 to 3.58) | 2.89 (1.06 to 7.91)* |
| NHW (Ref) |  |  |  |
|  |  |  |  |
| Health insurance ^c^ |  |  |  |
| Private (Ref) |  |  |  |
| Public | 1.08 (0.83 to 1.39) | 1.05 (0.74 to 1.50) | 1.15 (0.84 to 1.58) |
| None | 0.24 (0.11 to 0.53)*** | 0.34 (0.14 to 0.82)* | 0.11 (0.02 to 0.52)** |
|  |  |  |  |
| Income |  |  |  |
| <$25K (Ref) |  |  |  |
| $25K to $49,999 | 0.97 (0.75 to 1.25) | 0.88 (0.62 to 1.25) | 1.07 (0.75 to 1.53) |
| $50K to $74,999 | 0.83 (0.60 to 1.13) | 0.58 (0.39 to 0.86)** | 1.23 (0.78 to 1.93) |
| $75K to $99,999 | 1.23 (0.82 to 1.85) | 1.18 (0.71 to 1.95) | 1.21 (0.66 to 2.24) |
| $100K+ | 1.16 (0.77 to 1.75) | 1.01 (0.59 to 1.73) | 1.37 (0.74 to 2.53) |
|  |  |  |  |
| Education |  |  |  |
| Did not graduate HS (Ref) |  |  |  |
| Graduated HS | 1.11 (0.81 to 1.52) | 1.08 (0.70 to 1.66) | 1.20 (0.77 to 1.86) |
| Some college or technical school | 1.01 (0.73 to 1.40) | 1.16 (0.75 to 1.79) | 0.91 (0.58 to 1.43) |
| Graduated from college | 0.91 (0.61 to 1.35) | 0.97 (0.58 to 1.60) | 0.87 (0.49 to 1.55) |
|  |  |  |  |
| Smoking status |  |  |  |
| Currently smokes (Ref) |  |  |  |
| Formerly smoked | 1.04 (0.83 to 1.29) | 0.94 (0.70 to 1.25) | 1.18 (0.87 to 1.60) |
|  |  |  |  |
| Pack-year history |  |  |  |
| Years | 1.01 (1.00 to 1.01)*** | 1.01 (1.00 to 1.01)*** | 1.01 (1.00 to 1.01) |

^a^ Logistic regression analyses conducted where (*) p-value indicates p≤0.05 ; (**) indicates p≤0.01; (***) indicates p≤0.001. ^b^ NHO: non-Hispanic American Indian or Alaska Native, Asian, Native Hawaiian or Other Pacific Islander. ^c^ Private: Employer or private NGO; Public: Medicare, Medigap, Medicaid, CHIP, Military, Indian Health Service, Other gov't. Abbreviations: NHB, non-Hispanic Black; NHM, non-Hispanic Multiracial; NHO, non-Hispanic Other; NHW, non-Hispanic White; LCS, lung cancer screening.

e-Table 2a: Logistic regressions predicting associations between FMD & other covariates/pack-year history on LCS overall and by sex, BRFSS 2022 sample aged 50-79 years (Weighted)

| Variable | Odds of LCS [Odds ratio (95%CI)] ^a^ | | |
| --- | --- | --- | --- |
|  | Overall  (N=4,657,908) | Male  (N=2,607,088) | Female  (N=2,050,820) |
| Frequent mental distress (FMD) |  |  |  |
| Yes | 1.14 (0.87 to 1.48) | 1.18 (0.81 to 1.70) | 1.08 (0.78 to 1.49) |
| No (Ref) |  |  |  |
|  |  |  |  |
| Age |  |  |  |
| 50-64 years (Ref) |  |  |  |
| 65-79 years | 1.99 (1.59 to 2.49)*** | 2.27 (1.65 to 3.12)*** | 1.67 (1.26 to 2.27)*** |
|  |  |  |  |
| Sex |  |  |  |
| Male (Ref) |  |  |  |
| Female | 1.00 (0.81 to 1.22) | -- | -- |
|  |  |  |  |
| Race/ethnicity |  |  |  |
| Hispanic | 0.81 (0.43 to 1.55) | 0.88 (0.40 to 1.92) | 0.64 (0.22 to 1.88) |
| NHB | 1.09 (0.73 to 1.61) | 0.99 (0.62 to 1.60) | 1.25 (0.64 to 2.43) |
| NHM | 0.49 (0.29 to 0.84)** | 0.52 (0.25 to 1.05) | 0.44 (0.20 to 0.98)* |
| NHO ^b^ | 1.83 (0.87 to 3.85) | 1.28 (0.46 to 3.58) | 2.95 (1.07 to 8.09)* |
| NHW (Ref) |  |  |  |
|  |  |  |  |
| Health insurance  ^c^ |  |  |  |
| Private (Ref) |  |  |  |
| Public | 1.09 (0.85 to 1.40) | 1.05 (0.74 to 1.49) | 1.19 (0.87 to 1.64) |
| None | 0.24 (0.11 to 0.52)*** | 0.34 (0.14 to 0.81)* | 0.10 (0.02 to 0.50)** |
|  |  |  |  |
| Income |  |  |  |
| <$25K (Ref) |  |  |  |
| $25K to $49,999 | 0.97 (0.75 to 1.24) | 0.89 (0.63 to 1.26) | 1.05 (0.73 to 1.49) |
| $50K to $74,999 | 0.82 (0.60 to 1.11) | 0.59 (0.40 to 0.88)** | 1.18 (0.75 to 1.85) |
| $75K to $99,999 | 1.22 (0.81 to 1.83) | 1.20 (0.73 to 1.98) | 1.18 (0.64 to 2.16) |
| $100K+ | 1.15 (0.76 to 1.72) | 1.04 (0.61 to 1.78) | 1.31 (0.71 to 2.43) |
|  |  |  |  |
| Education |  |  |  |
| Did not graduate HS (Ref) |  |  |  |
| Graduated HS | 1.11 (0.81 to 1.52) | 1.08 (0.70 to 1.66) | 1.21 (0.77 to 1.89) |
| Some college or technical school | 1.04 (0.75 to 1.43) | 1.16 (0.75 to 1.79) | 0.95 (0.61 to 1.50) |
| Graduated from college | 0.93 (0.63 to 1.37) | 0.98 (0.59 to 1.61) | 0.91 (0.51 to 1.61) |
|  |  |  |  |
| Smoking status |  |  |  |
| Currently smokes (Ref) |  |  |  |
| Formerly smoked | 1.04 (0.84 to 1.29) | 0.94 (0.71 to 1.26) | 1.17 (0.86 to 1.57) |
|  |  |  |  |
| Pack-year history |  |  |  |
| Years | 1.01 (1.00 to 1.01)*** | 1.01 (1.00 to 1.01)*** | 1.01 (1.00 to 1.01)* |

^a^ Logistic regression analyses conducted where (*) p-value indicates p≤0.05 ; (**) indicates p≤0.01; (***) indicates p≤0.001. ^b^ NHO: non-Hispanic American Indian or Alaska Native, Asian, Native Hawaiian or Other Pacific Islander. ^c^ Private: Employer or private NGO; Public: Medicare, Medigap, Medicaid, CHIP, Military, Indian Health Service, Other gov't. Abbreviations: NHB, non-Hispanic Black; NHM, non-Hispanic Multiracial; NHO, non-Hispanic Other; NHW, non-Hispanic White; LCS, lung cancer screening.

e-Table 3a: Logistic regressions predicting associations between depressive disorder history & other covariates/pack-year history on LCS overall and by race/ethnicity, BRFSS 2022 sample aged 50-79 years (Weighted)

| Variable | Odds of LCS (Odds ratio [95%CI])  ^a^ | | | | | |
| --- | --- | --- | --- | --- | --- | --- |
|  | Overall  (N=4,641,820) | NHW  (N=3,555,440) | NHB (N=420,028) | Hispanic (N=291,580) | NHM  (N=167,358) | NHO (N=137,192) |
| Depressive disorder history |  |  |  |  |  |  |
| Yes | 1.15 (0.90 to 1.47) | 1.14 (0.88 to 1.46) | 0.84 (0.35 to 2.05) | 0.66 (0.16 to 2.79) | 0.62 (0.17 to 2.27) | 6.73 (1.67 to 27.13)** |
| No (Ref) |  |  |  |  |  |  |
|  |  |  |  |  |  |  |
| Age |  |  |  |  |  |  |
| 50-64 years (Ref) |  |  |  |  |  |  |
| 65-79 years | 2.04 (1.63 to 2.55)*** | 2.02 (1.62 to 2.51)*** | 2.23 (1.11 to 4.48)* | 0.71 (0.25 to 2.03) | 2.18 (0.56 to 8.44) | 2.83 (0.66 to 12.01) |
|  |  |  |  |  |  |  |
| Sex |  |  |  |  |  |  |
| Male (Ref) |  |  |  |  |  |  |
| Female | 0.98 (0.80 to 1.21) | 0.94 (0.76 to 1.17) | 1.43 (0.65 to 3.14) | 0.37 (0.12 to 1.08) | 0.49 (0.14 to 1.66) | 1.98 (0.59 to 6.60) |
|  |  |  |  |  |  |  |
| Race/ethnicity |  |  |  |  |  |  |
| Hispanic | 0.81 (0.42 to 1.55) | -- | -- | -- | -- | -- |
| NHB | 1.09 (0.74 to 1.62) | -- | -- | -- | -- | -- |
| NHM | 0.47 (0.27 to 0.81)** | -- | -- | -- | -- | -- |
| NHO ^b^ | 1.85 (0.88 to 3.90) | -- | -- | -- | -- | -- |
| NHW (Ref) |  |  |  |  |  |  |
|  |  |  |  |  |  |  |
| Health insurance  ^c^ |  |  |  |  |  |  |
| Private (Ref) |  |  |  |  |  |  |
| Public | 1.08 (0.83 to 1.39) | 1.11 (0.86 to 1.43) | 1.17 (0.50 to 2.77) | 0.47 (0.13 to 1.76) | 1.76 (0.30 to 10.26) | 2.32 (0.50 to 10.78) |
| None | 0.24 (0.11 to 0.53)*** | 1.17 (0.06 to 0.46)*** | 2.12 (0.55 to 8.19) | to to | to to |  |
|  |  |  |  |  |  |  |
| Income |  |  |  |  |  |  |
| <$25K (Ref) |  |  |  |  |  |  |
| $25K to $49,999 | 0.97 (0.75 to 1.25) | 0.96 (0.74 to 1.24) | 0.81 (0.35 to 1.86) | 0.78 (0.17 to 3.57) | 0.18 (0.04 to 0.76)* | 10.45 (2.10 to 52.06)** |
| $50K to $74,999 | 0.83 (0.60 to 1.13) | 0.94 (0.69 to 1.29) | 0.31 (0.08 to 1.18) | 0.25 (0.05 to 1.23) | 0.26 (0.03 to 2.05) | to to |
| $75K to $99,999 | 1.23 (0.82 to 1.85) | 1.25 (0.84 to 1.87) | 3.53 (0.89 to 13.96) | 0.08 (0.01 to 0.90)* | 0.23 (0.02 to 2.48) | 2.69 (0.23 to 31.61) |
| $100K+ | 1.16 (0.77 to 1.75) | 1.18 (0.78 to 1.80) | 0.51 (0.13 to 1.99) | 0.02 (0.00 to 0.17)*** | 0.91 (0.90 to 9.25) | 29.82 (3.47 to 256.16)** |
|  |  |  |  |  |  |  |
| Education |  |  |  |  |  |  |
| Did not graduate HS (Ref) |  |  |  |  |  |  |
| Graduated HS | 1.11 (0.81 to 1.52) | 1.18 (0.84 to 1.65) | 0.39 (0.15 to 1.00)* | 2.27 (0.46 to 11.25) | 0.46 (0.10 to 2.20) | 1.31 (0.19 to 9.14) |
| Some college or technical school | 1.01 (0.73 to 1.40) | 0.98 (0.69 to 1.38) | 0.55 (0.20 to 1.50) | 5.32 (1.35 to 20.94)* | 1.68 (0.35 to 7.97) | 1.18 (0.20 to 7.00) |
| Graduated from college | 0.91 (0.61 to 1.35) | 0.85 (0.58 to 1.26) | 0.52 (0.16 to 1.65) | 9.40 (1.02 to 86.42)* | 0.85 (0.13 to 5.49) | 1.79 (0.16 to 19.91) |
|  |  |  |  |  |  |  |
| Smoking status |  |  |  |  |  |  |
| Currently smokes (Ref) |  |  |  |  |  |  |
| Formerly smoked | 1.04 (0.83 to 1.29) | 1.01 (0.81 to 1.24) | 1.25 (0.57 to 2.71) | 1.47 (0.45 to 4.81) | 3.74 (1.33 to 10.53)** | 0.53 (0.14 to 2.05) |
|  |  |  |  |  |  |  |
| Pack-year history |  |  |  |  |  |  |
| Years | 1.01 (1.00 to 1.01)*** | 1.00 (1.00 to 1.01)** | 1.01 (1.00 to 1.03) | 1.01 (0.99 to 1.04) | 1.01 (0.99 to 1.03) | 1.04 (1.01 to 1.06)** |
|  |  |  |  |  |  |  |

^a^ Logistic regression analyses conducted where (*) p-value indicates p≤0.05 ; (**) indicates p≤0.01; (***) indicates p≤0.001. ^b^ NHO: non-Hispanic American Indian or Alaska Native, Asian, Native Hawaiian or Other Pacific Islander. ^c^ Private: Employer or private NGO; Public: Medicare, Medigap, Medicaid, CHIP, Military, Indian Health Service, Other gov't. Abbreviations: NHB, non-Hispanic Black; NHM, non-Hispanic Multiracial; NHO, non-Hispanic Other; NHW, non-Hispanic White; LCS, lung cancer screening.

e-Table 4a: Logistic regressions predicting associations between FMD & other covariates/pack-year history on LCS overall and by race/ethnicity, BRFSS 2022 sample aged 50-79 years (Weighted)

| Variable | Odds of LCS (Odds ratio [95%CI])  ^a^ | | | | | |
| --- | --- | --- | --- | --- | --- | --- |
|  | Overall (N=4,657,908) | NHW  (N=3,568,022) | NHB (N=421,203) | Hispanic (N=291,580) | NHM  (N=168,827) | NHO (N=138,053) |
| Frequent mental distress (FMD) |  |  |  |  |  |  |
| Yes | 1.14 (0.87 to 1.48) | 1.03 (0.79 to 1.36) | 1.10 (0.45 to 2.65) | 1.94 (0.58 to 6.54) | 0.97 (0.24 to 3.86) | 3.41 (0.90 to 12.95) |
| No (Ref) |  |  |  |  |  |  |
|  |  |  |  |  |  |  |
| Age |  |  |  |  |  |  |
| 50-64 years (Ref) |  |  |  |  |  |  |
| 65-79 years | 1.99 (1.59 to 2.49)*** | 1.96 (1.57 to 2.45)*** | 2.30 (1.13 to 4.70)* | 0.84 (0.29 to 2.40) | 2.11 (0.65 to 6.89) | 1.91 (0.48 to 7.60) |
|  |  |  |  |  |  |  |
| Sex |  |  |  |  |  |  |
| Male (Ref) |  |  |  |  |  |  |
| Female | 1.00 (0.81 to 1.22) | 0.96 (0.78 to 1.18) | 1.43 (0.64 to 3.20) | 0.35 (0.13 to 0.92)* | 0.52 (0.15 to 1.78) | 2.53 (0.76 to 8.39) |
|  |  |  |  |  |  |  |
| Race/ethnicity |  |  |  |  |  |  |
| Hispanic | 0.81 (0.43 to 1.55) | -- | -- | -- | -- | -- |
| NHB | 1.09 (0.73 to 1.61) | -- | -- | -- | -- | -- |
| NHM | 0.49 (0.29 to 0.84)** | -- | -- | -- | -- | -- |
| NHO ^b^ | 1.83 (0.87 to 3.85) | -- | -- | -- | -- | -- |
| NHW (Ref) |  |  |  |  |  |  |
|  |  |  |  |  |  |  |
| Health insurance  ^c^ |  |  |  |  |  |  |
| Private (Ref) |  |  |  |  |  |  |
| Public | 1.09 (0.85 to 1.40) | 1.12 (0.87 to 1.45) | 1.14 (0.47 to 2.74) | 0.52 (0.14 to 1.96) | 1.60 (0.30 to 8.48) | 3.03 (0.65 to 14.16) |
| None | 0.24 (0.11 to 0.52)*** | 0.17 (0.06 to 0.45)*** | 2.11 (0.55 to 8.11) | to to | to to | to to |
|  |  |  |  |  |  |  |
| Income |  |  |  |  |  |  |
| <$25K (Ref) |  |  |  |  |  |  |
| $25K to $49,999 | 0.97 (0.75 to 1.24) | 0.96 (0.74 to 1.23) | 0.82 (0.36 to 1.87) | 0.94 (0.21 to 4.22) | 0.16 (0.04 to 0.71)* | 13.58 (2.40 to 76.94)** |
| $50K to $74,999 | 0.82 (0.60 to 1.11) | 0.93 (0.68 to 1.28) | 0.31 (0.08 to 1.20) | 0.32 (0.07 to 1.53) | 0.24 (0.03 to 1.80) | to to |
| $75K to $99,999 | 1.22 (0.81 to 1.83) | 1.24 (0.84 to 1.83) | 3.59 (0.87 to 14.83) | 0.09 (0.01 to 1.17) | 0.23 (0.02 to 2.15) | 1.05 (0.07 to 16.19) |
| $100K+ | 1.15 (0.76 to 1.72) | 1.16 (0.77 to 1.75) | 0.53 (0.14 to 2.07) | 0.02 (0.00 to 0.23)** | 0.95 (0.10 to 8.72) | 30.08 (3.25 to 278.75)** |
|  |  |  |  |  |  |  |
| Education |  |  |  |  |  |  |
| Did not graduate HS (Ref) |  |  |  |  |  |  |
| Graduated HS | 1.11 (0.81 to 1.52) | 1.18 (0.84 to 1.65) | 0.41 (0.17 to 1.01)* | 2.91 (0.54 to 15.57) | 0.38 (0.07 to 2.11) | 0.70 (0.10 to 4.91) |
| Some college or technical school | 1.04 (0.75 to 1.43) | 0.99 (0.70 to 1.41) | 0.57 (0.21 to 1.53) | 5.18 (1.38 to 19.37)* | 1.93 (0.42 to 8.87) | 0.73 (0.11 to 5.03) |
| Graduated from college | 0.93 (0.63 to 1.37) | 0.87 (0.59 to 1.28) | 0.54 (0.17 to 1.69) | 12.52 (1.34 to 116.83)* | 0.76 (0.13 to 4.35) | 0.97 (0.08 to 11.77) |
|  |  |  |  |  |  |  |
| Smoking status |  |  |  |  |  |  |
| Currently smokes (Ref) |  |  |  |  |  |  |
| Formerly smoked | 1.04 (0.84 to 1.29) | 1.00 (0.81 to 1.24) | 1.25 (0.56 to 2.79) | 1.34 (0.39 to 4.64) | 3.44 (1.27 to 9.32)* | 0.63 (0.19 to 2.12) |
|  |  |  |  |  |  |  |
| Pack-year history |  |  |  |  |  |  |
| Years | 1.01 (1.00 to 1.01)*** | 1.01 (1.00 to 1.01)** | 1.01 (1.00 to 1.03) | 1.01 (0.98 to 1.04) | 1.01 (0.99 to 1.03) | 1.04 (1.01 to 1.07)** |
|  |  |  |  |  |  |  |

^a^ Logistic regression analyses conducted where (*) p-value indicates p≤0.05 ; (**) indicates p≤0.01; (***) indicates p≤0.001. ^b^ NHO: non-Hispanic American Indian or Alaska Native, Asian, Native Hawaiian or Other Pacific Islander. ^c^ Private: Employer or private NGO; Public: Medicare, Medigap, Medicaid, CHIP, Military, Indian Health Service, Other gov't. Abbreviations: NHB, non-Hispanic Black; NHM, non-Hispanic Multiracial; NHO, non-Hispanic Other; NHW, non-Hispanic White; LCS, lung cancer screening.

e-Table 1b: Logistic regressions predicting associations between depressive disorder history & other covariates/cigarettes per day on LCS overall and by sex, BRFSS 2022 sample aged 50-79 years (Weighted)

| Variable | Odds of LCS [Odds ratio (95%CI)] ^a^ | | |
| --- | --- | --- | --- |
|  | Overall  (N=4,641,820) | Male  (N=2,598,051) | Female  (N=2,043,769) |
| Depressive disorder history |  |  |  |
| Yes | 1.16 (0.91 to 1.47) | 0.97 (0.66 to 1.41) | 1.33 (0.99 to 1.80) |
| No (Ref) |  |  |  |
|  |  |  |  |
| Age |  |  |  |
| 50-64 years (Ref) |  |  |  |
| 65-79 years | 2.18 (1.74 to 2.73)*** | 2.47 (1.79 to 3.42)*** | 1.87 (1.41 to 2.48)*** |
|  |  |  |  |
| Sex |  |  |  |
| Male (Ref) |  |  |  |
| Female | 0.97 (0.79 to 1.20) | -- | -- |
|  |  |  |  |
| Race/ethnicity |  |  |  |
| Hispanic | 0.78 (0.41 to 1.50) | 0.82 (0.37 to 1.81) | 0.66 (0.23 to 1.93) |
| NHB | 1.04 (0.70 to 1.55) | 0.92 (0.57 to 1.48) | 1.27 (0.65 to 2.48) |
| NHM | 0.46 (0.27 to 0.81)** | 0.52 (0.25 to 1.06) | 0.36 (0.15 to 0.87)* |
| NHO ^b^ | 1.84 (0.86 to 3.91) | 1.25 (0.45 to 3.48) | 2.94 (1.06 to 8.15)* |
| NHW (Ref) |  |  |  |
|  |  |  |  |
| Health insurance  ^c^ |  |  |  |
| Private (Ref) |  |  |  |
| Public | 1.09 (0.85 to 1.41) | 1.08 (0.76 to 1.52) | 1.16 (0.85 to 1.60) |
| None | 0.24 (0.11 to 0.54)*** | 0.34 (0.14 to 0.83)* | 0.11 (0.02 to 0.52)** |
|  |  |  |  |
| Income |  |  |  |
| <$25K (Ref) |  |  |  |
| $25K to $49,999 | 0.96 (0.75 to 1.24) | 0.88 (0.62 to 1.25) | 1.06 (0.74 to 1.51) |
| $50K to $74,999 | 0.82 (0.60 to 1.11) | 0.58 (0.39 to 0.86)** | 1.22 (0.78 to 1.91) |
| $75K to $99,999 | 1.21 (0.81 to 1.82) | 1.16 (0.70 to 1.92) | 1.21 (0.66 to 2.22) |
| $100K+ | 1.16 (0.77 to 1.75) | 1.01 (0.59 to 1.72) | 1.36 (0.74 to 2.53) |
|  |  |  |  |
| Education |  |  |  |
| Did not graduate HS (Ref) |  |  |  |
| Graduated HS | 1.07 (0.78 to 1.47) | 1.02 (0.66 to 1.58) | 1.18 (0.76 to 1.84) |
| Some college or technical school | 0.99 (0.71 to 1.37) | 1.10 (0.71 to 1.71) | 0.90 (0.58 to 1.41) |
| Graduated from college | 0.88 (0.59 to 1.31) | 0.92 (0.55 to 1.53) | 0.86 (0.48 to 1.51) |
|  |  |  |  |
| Smoking status |  |  |  |
| Currently smokes (Ref) |  |  |  |
| Formerly smoked | 1.03 (0.82 to 1.28) | 0.94 (0.70 to 1.26) | 1.16 (0.85 to 1.58) |
|  |  |  |  |
| Cigarettes per day, lifetime smokers |  |  |  |
| # cigs/day | 1.01 (1.00 to 1.02)* | 1.01 (1.00 to 1.02) | 1.01 (1.00 to 1.02) |

^a^ Logistic regression analyses conducted where (*) p-value indicates p≤0.05 ; (**) indicates p≤0.01; (***) indicates p≤0.001. ^b^ NHO: non-Hispanic American Indian or Alaska Native, Asian, Native Hawaiian or Other Pacific Islander. ^c^ Private: Employer or private NGO; Public: Medicare, Medigap, Medicaid, CHIP, Military, Indian Health Service, Other gov't. Abbreviations: NHB, non-Hispanic Black; NHM, non-Hispanic Multiracial; NHO, non-Hispanic Other; NHW, non-Hispanic White; LCS, lung cancer screening.

e-Table 2b: Logistic regressions predicting associations between FMD & other covariates/cigarettes per day on LCS overall and by sex, BRFSS 2022 sample aged 50-79 years (Weighted)

| Variable | Odds of LCS [Odds ratio (95%CI)] ^a^ | | |
| --- | --- | --- | --- |
|  | Overall  (N=4,657,908) | Male  (N=2,607,088) | Female  (N=2,050,820) |
| Frequent mental distress (FMD) |  |  |  |
| Yes | 1.14 (0.87 to 1.48) | 1.17 (0.81 to 1.70) | 1.08 (0.78 to 1.49) |
| No (Ref) |  |  |  |
|  |  |  |  |
| Age |  |  |  |
| 50-64 years (Ref) |  |  |  |
| 65-79 years | 2.13 (1.70 to 2.67)*** | 2.47 (1.79 to 3.41)*** | 1.76 (1.33 to 2.34)*** |
|  |  |  |  |
| Sex |  |  |  |
| Male (Ref) |  |  |  |
| Female | 0.98 (0.80 to 1.21) | -- | -- |
|  |  |  |  |
| Race/ethnicity |  |  |  |
| Hispanic | 0.79 (0.41 to 1.50) | 0.83 (0.38 to 1.81) | 0.64 (0.22 to 1.87) |
| NHB | 1.04 (0.70 to 1.53) | 0.92 (0.58 to 1.48) | 1.22 (0.62 to 2.37) |
| NHM | 0.49 (0.29 to 0.84)** | 0.51 (0.25 to 1.04) | 0.44 (0.19 to 0.99)* |
| NHO ^b^ | 1.82 (0.85 to 3.86) | 1.24 (0.44 to 3.49) | 2.99 (1.07 to 8.34)* |
| NHW (Ref) |  |  |  |
|  |  |  |  |
| Health insurance  ^c^ |  |  |  |
| Private (Ref) |  |  |  |
| Public | 1.11 (0.86 to 1.42) | 1.07 (0.76 to 1.52) | 1.20 (0.87 to 1.65) |
| None | 0.24 (0.11 to 0.53)*** | 0.34 (0.14 to 0.82)* | 0.10 (0.02 to 0.50)** |
|  |  |  |  |
| Income |  |  |  |
| <$25K (Ref) |  |  |  |
| $25K to $49,999 | 0.96 (0.75 to 1.23) | 0.89 (0.63 to 1.26) | 1.04 (0.73 to 1.47) |
| $50K to $74,999 | 0.81 (0.60 to 1.11) | 0.59 (0.40 to 0.88)** | 1.17 (0.75 to 1.83) |
| $75K to $99,999 | 1.20 (0.80 to 1.80) | 1.18 (0.72 to 1.94) | 1.18 (0.64 to 2.15) |
| $100K+ | 1.14 (0.76 to 1.71) | 1.04 (0.61 to 1.76) | 1.31 (0.71 to 2.43) |
|  |  |  |  |
| Education |  |  |  |
| Did not graduate HS (Ref) |  |  |  |
| Graduated HS | 1.07 (0.78 to 1.48) | 1.03 (0.66 to 1.59) | 1.19 (0.76 to 1.86) |
| Some college or technical school | 1.01 (0.73 to 1.40) | 1.11 (0.71 to 1.72) | 0.94 (0.60 to 1.48) |
| Graduated from college | 0.89 (0.60 to 1.33) | 0.92 (0.55 to 1.54) | 0.89 (0.50 to 1.58) |
|  |  |  |  |
| Smoking status |  |  |  |
| Currently smokes (Ref) |  |  |  |
| Formerly smoked | 1.03 (0.83 to 1.28) | 0.94 (0.70 to 1.26) | 1.15 (0.84 to 1.56) |
|  |  |  |  |
| Cigarettes per day, lifetime smokers |  |  |  |
| # cigs/day | 1.01 (1.00 to 1.02)* | 1.01 (1.00 to 1.02) | 1.01 (1.00 to 1.02) |

^a^ Logistic regression analyses conducted where (*) p-value indicates p≤0.05 ; (**) indicates p≤0.01; (***) indicates p≤0.001. ^b^ NHO: non-Hispanic American Indian or Alaska Native, Asian, Native Hawaiian or Other Pacific Islander. ^c^ Private: Employer or private NGO; Public: Medicare, Medigap, Medicaid, CHIP, Military, Indian Health Service, Other gov't. Abbreviations: NHB, non-Hispanic Black; NHM, non-Hispanic Multiracial; NHO, non-Hispanic Other; NHW, non-Hispanic White; LCS, lung cancer screening.

e-Table 3b: Logistic regressions predicting associations between depressive disorder history & other covariates/cigarettes per day on LCS overall and by race/ethnicity, BRFSS 2022 sample aged 50-79 years (Weighted)

| Variable | Odds of LCS (Odds ratio [95%CI])  ^a^ | | | | | |
| --- | --- | --- | --- | --- | --- | --- |
|  | Overall  (N=4,641,820) | NHW  (N=3,555,440) | NHB (N=420,028) | Hispanic (N=291,580) | NHM  (N=167,358) | NHO (N=137,192) |
| Depressive disorder history |  |  |  |  |  |  |
| Yes | 1.16 (0.91 to 1.47) | 1.14 (0.89 to 1.46) | 0.88 (0.36 to 2.14) | 0.74 (0.19 to 2.86) | 0.61 (0.17 to 2.21) | 6.39 (1.63 to 25.05)** |
| No (Ref) |  |  |  |  |  |  |
|  |  |  |  |  |  |  |
| Age |  |  |  |  |  |  |
| 50-64 years (Ref) |  |  |  |  |  |  |
| 65-79 years | 2.18 (1.74 to 2.73)*** | 2.12 (1.70 to 2.65)*** | 2.46 (1.23 to 4.92)** | 0.85 (0.26 to 2.74) | 2.52 (0.68 to 9.35) | 4.58 (1.07 to 19.61)* |
|  |  |  |  |  |  |  |
| Sex |  |  |  |  |  |  |
| Male (Ref) |  |  |  |  |  |  |
| Female | 0.97 (0.79 to 1.20) | 0.93 (0.76 to 1.15) | 1.41 (0.65 to 3.07) | 0.36 (0.12 to 1.08) | 0.49 (0.14 to 1.65) | 2.39 (0.72 to 7.92) |
|  |  |  |  |  |  |  |
| Race/ethnicity |  |  |  |  |  |  |
| Hispanic | 0.78 (0.41 to 1.50) | -- | -- | -- | -- | -- |
| NHB | 1.04 (0.70 to 1.55) | -- | -- | -- | -- | -- |
| NHM | 0.46 (0.27 to 0.81)** | -- | -- | -- | -- | -- |
| NHO ^b^ | 1.84 (0.86 to 3.91) | -- | -- | -- | -- | -- |
| NHW (Ref) |  |  |  |  |  |  |
|  |  |  |  |  |  |  |
| Health insurance  ^c^ |  |  |  |  |  |  |
| Private (Ref) |  |  |  |  |  |  |
| Public | 1.09 (0.85 to 1.41) | 1.13 (0.87 to 1.46) | 1.18 (0.50 to 2.80) | 0.45 (0.12 to 1.66) | 1.74 (0.29 to 10.30) | 2.29 (0.48 to 10.94) |
| None | 0.24 (0.11 to 0.54)*** | 0.17 (0.06 to 0.46)*** | 2.15 (0.56 to 8.27) | -- | -- | -- |
|  |  |  |  |  |  |  |
| Income |  |  |  |  |  |  |
| <$25K (Ref) |  |  |  |  |  |  |
| $25K to $49,999 | 0.96 (0.75 to 1.24) | 0.95 (0.74 to 1.23) | 0.81 (0.36 to 1.83) | 0.73 (0.16 to 3.40) | 0.17 (0.04 to 0.73)* | 10.53 (2.05 to 54.13)** |
| $50K to $74,999 | 0.82 (0.60 to 1.11) | 0.93 (0.68 to 1.28) | 0.31 (0.08 to 1.21) | 0.24 (0.05 to 1.18) | 0.23 (0.03 to 1.84) | -- |
| $75K to $99,999 | 1.21 (0.81 to 1.82) | 1.24 (0.83 to 1.85) | 3.43 (0.87 to 13.57) | 0.07 (0.01 to 0.79)* | 0.22 (0.02 to 2.38) | 2.51 (0.21 to 29.71) |
| $100K+ | 1.16 (0.77 to 1.75) | 1.18 (0.78 to 1.79) | 0.48 (0.12 to 1.96) | 0.12 (0.00 to 0.17)*** | 0.81 (0.08 to 8.20) | 28.62 (3.18 to 257.32)** |
|  |  |  |  |  |  |  |
| Education |  |  |  |  |  |  |
| Did not graduate HS (Ref) |  |  |  |  |  |  |
| Graduated HS | 1.07 (0.78 to 1.47) | 1.14 (0.81 to 1.62) | 0.37 (0.15 to 0.95)* | 2.41 (0.47 to 12.41) | 0.43 (0.09 to 2.06) | 0.86 (0.14 to 5.37) |
| Some college or technical school | 0.99 (0.71 to 1.37) | 0.96 (0.67 to 1.36) | 0.53 (0.20 to 1.43) | 5.24 (1.30 to 21.04)* | 1.51 (0.33 to 6.85) | 0.93 (0.18 to 4.88) |
| Graduated from college | 0.88 (0.59 to 1.31) | 0.83 (0.56 to 1.23) | 0.51 (0.16 to 1.60) | 10.30 (1.10 to 96.66)* | 0.82 (0.13 to 5.13) | 1.33 (0.16 to 11.47) |
|  |  |  |  |  |  |  |
| Smoking status |  |  |  |  |  |  |
| Currently smokes (Ref) |  |  |  |  |  |  |
| Formerly smoked | 1.03 (0.82 to 1.28) | 1.01 (0.81 to 1.25) | 1.19 (0.53 to 2.67) | 1.56 (0.46 to 5.25) | 1.03 (0.98 to 1.07) | 0.45 (0.11 to 1.78) |
|  |  |  |  |  |  |  |
| Cigarettes per day, lifetime smokers |  |  |  |  |  |  |
| # cigs/day | 1.01 (1.00 to 1.02)* | 1.00 (1.00 to 1.01) | 1.02 (0.99 to 1.06) | 1.00 (0.94 to 1.07) | 1.03 (0.98 to 1.07) | 1.08 (1.01 to 1.15)* |
|  |  |  |  |  |  |  |

^a^ Logistic regression analyses conducted where (*) p-value indicates p≤0.05 ; (**) indicates p≤0.01; (***) indicates p≤0.001. ^b^ NHO: non-Hispanic American Indian or Alaska Native, Asian, Native Hawaiian or Other Pacific Islander. ^c^ Private: Employer or private NGO; Public: Medicare, Medigap, Medicaid, CHIP, Military, Indian Health Service, Other gov't. Abbreviations: NHB, non-Hispanic Black; NHM, non-Hispanic Multiracial; NHO, non-Hispanic Other; NHW, non-Hispanic White; LCS, lung cancer screening.

e-Table 4b: Logistic regressions predicting associations between FMD & other covariates/cigarettes per day on LCS overall and by race/ethnicity, BRFSS 2022 sample aged 50-79 years (Weighted)

| Variable | Odds of LCS (Odds ratio [95%CI])  ^a^ | | | | | |
| --- | --- | --- | --- | --- | --- | --- |
|  | Overall (N=4,657,908) | NHW  (N=3,568,022) | NHB (N=421,203) | Hispanic (N=291,580) | NHM  (N=168,827) | NHO (N=138,053) |
| Frequent mental distress (FMD) |  |  |  |  |  |  |
| Yes | 1.14 (0.87 to 1.48) | 1.03 (0.79 to 1.36) | 1.09 (0.45 to 2.65) | 1.93 (0.58 to 6.38) | 0.93 (0.23 to 3.73) | 3.32 (0.90 to 12.19) |
| No (Ref) |  |  |  |  |  |  |
|  |  |  |  |  |  |  |
| Age |  |  |  |  |  |  |
| 50-64 years (Ref) |  |  |  |  |  |  |
| 65-79 years | 2.13 (1.70 to 2.67)*** | 2.07 (1.65 to 2.59)*** | 2.54 (1.25 to 5.17)** | 0.95 (0.29 to 3.16) | 2.30 (0.70 to 7.54) | 3.19 (0.83 to 12.28) |
|  |  |  |  |  |  |  |
| Sex |  |  |  |  |  |  |
| Male (Ref) |  |  |  |  |  |  |
| Female | 0.98 (0.80 to 1.21) | 0.95 (0.77 to 1.17) | 1.41 (0.63 to 3.12) | 0.34 (0.12 to 0.92)* | 0.52 (0.15 to 1.77) | 2.96 (0.89 to 9.82) |
|  |  |  |  |  |  |  |
| Race/ethnicity |  |  |  |  |  |  |
| Hispanic | 0.79 (0.41 to 1.50) | -- | -- | -- | -- | -- |
| NHB | 1.04 (0.70 to 1.53) | -- | -- | -- | -- | -- |
| NHM | 0.49 (0.29 to 0.84)** | -- | -- | -- | -- | -- |
| NHO ^b^ | 1.82 (0.85 to 3.86) | -- | -- | -- | -- | -- |
| NHW (Ref) |  |  |  |  |  |  |
|  |  |  |  |  |  |  |
| Health insurance  ^c^ |  |  |  |  |  |  |
| Private (Ref) |  |  |  |  |  |  |
| Public | 1.11 (0.86 to 1.42) | 1.14 (0.89 to 1.47) | 1.16 (0.48 to 2.79) | 0.48 (0.13 to 1.83) | 1.60 (0.30 to 8.58) | 2.94 (0.62 to 13.94) |
| None | 0.24 (0.11 to 0.53)*** | 0.17 (0.06 to 0.45)*** | 2.15 (0.56 to 8.21) | -- | -- | -- |
|  |  |  |  |  |  |  |
| Income |  |  |  |  |  |  |
| <$25K (Ref) |  |  |  |  |  |  |
| $25K to $49,999 | 0.96 (0.75 to 1.23) | 0.95 (0.74 to 1.22) | 0.82 (0.36 to 1.84) | 0.87 (0.19 to 4.01) | 0.15 (0.03 to 0.71)* | 13.78 (2.41 to 78.91)** |
| $50K to $74,999 | 0.81 (0.60 to 1.11) | 0.93 (0.68 to 1.27) | 0.31 (0.08 to 1.22) | 0.31 (0.07 to 1.48) | 0.21 (0.03 to 1.59) | -- |
| $75K to $99,999 | 1.20 (0.80 to 1.80) | 1.23 (0.83 to 1.81) | 3.47 (0.85 to 14.22) | 0.08 (0.01 to 1.01)* | 0.21 (0.02 to 2.06) | 1.03 (0.07 to 14.70) |
| $100K+ | 1.14 (0.76 to 1.71) | 1.16 (0.77 to 1.74) | 0.50 (0.12 to 2.01) | 0.02 (0.00 to 0.22)** | 0.86 (0.10 to 7.59) | 28.53 (3.01 to 270.08)** |
|  |  |  |  |  |  |  |
| Education |  |  |  |  |  |  |
| Did not graduate HS (Ref) |  |  |  |  |  |  |
| Graduated HS | 1.07 (0.78 to 1.48) | 1.15 (0.81 to 1.62) | 0.39 (0.16 to 0.96)* | 3.07 (0.56 to 16.99) | 0.36 (0.06 to 2.01) | 0.47 (0.07 to 3.18) |
| Some college or technical school | 1.01 (0.73 to 1.40) | 0.97 (0.68 to 1.39) | 0.55 (0.21 to 1.45) | 5.13 (1.33 to 19.81) | 1.71 (0.38 to 7.77) | 0.60 (0.10 to 3.65) |
| Graduated from college | 0.89 (0.60 to 1.33) | 0.84 (0.57 to 1.25) | 0.53 (0.17 to 1.63) | 13.38 (1.40 to 127.67)* | 0.72 (0.13 to 4.06) | 0.74 (0.08 to 7.29) |
|  |  |  |  |  |  |  |
| Smoking status |  |  |  |  |  |  |
| Currently smokes (Ref) |  |  |  |  |  |  |
| Formerly smoked | 1.03 (0.83 to 1.28) | 1.00 (0.81 to 1.24) | 1.19 (0.52 to 2.75) | 1.45 (0.39 to 5.44) | 3.65 (1.35 to 9.91)** | 0.52 (0.15 to 1.83) |
|  |  |  |  |  |  |  |
| Cigarettes per day, lifetime smokers |  |  |  |  |  |  |
| # cigs/day | 1.01 (1.00 to 1.02)* | 1.00 (1.00 to 1.01) | 1.02 (0.99 to 1.06) | 1.00 (0.93 to 1.07) | 1.01 (0.97 to 1.06) | 1.08 (1.01 to 1.16)** |
|  |  |  |  |  |  |  |

^a^ Logistic regression analyses conducted where (*) p-value indicates p≤0.05 ; (**) indicates p≤0.01; (***) indicates p≤0.001. ^b^ NHO: non-Hispanic American Indian or Alaska Native, Asian, Native Hawaiian or Other Pacific Islander. ^c^ Private: Employer or private NGO; Public: Medicare, Medigap, Medicaid, CHIP, Military, Indian Health Service, Other gov't. Abbreviations: NHB, non-Hispanic Black; NHM, non-Hispanic Multiracial; NHO, non-Hispanic Other; NHW, non-Hispanic White; LCS, lung cancer screening.
